# Remote electrical neuromodulation (REN) in the acute treatment of migraine: a comparison with usual care and acute migraine medications

**DOI:** 10.1186/s10194-019-1033-9

**Published:** 2019-07-22

**Authors:** Alan M. Rapoport, Jo H. Bonner, Tamar Lin, Dagan Harris, Yaron Gruper, Alon Ironi, Robert P. Cowan

**Affiliations:** 1 The David Geffen School of Medicine, UCLA, California, Los Angeles USA; 2 Mercy Clinic Neurology & Headache Cente, St. Louis, MO USA; 3 Theranica Bio-Electronics LTD, Netanya, Israel; 4 School of medicine, Stanford University, Stanford, CA USA

**Keywords:** Remote electrical neuromodulation, Migraine, Headache, Conditioned pain modulation, Neuromodulation, Acute treatment, Non-pharmacological treatment

## Abstract

**Background:**

There is a significant unmet need for new, effective and well tolerated acute migraine treatments. A recent study has demonstrated that a novel remote electrical neuromodulation (REN) treatment provides superior clinically meaningful pain relief with a low rate of device-related adverse events. The results reported herein compare the efficacy of REN with current standard of care in the acute treatments of migraine.

**Methods:**

We performed a post-hoc analysis on a subgroup of participants with migraine from a randomized, double-blind, parallel-group, sham-controlled, multicenter study on acute care. The original study included a 2–4 weeks run-in phase, in which migraine attacks were treated according to patient preference (i.e., usual care) and reported in an electronic diary; next, participants entered a double-blind treatment phase in which they treated the attacks with an active or sham device. The efficacy of REN was compared to the efficacy of usual care or pharmacological treatments in the run-in phase in a within-subject design that included participants who treated at least one attack with the active REN device and reported pain intensity at 2 h post-treatment.

**Results:**

Of the 252 patients randomized, there were 99 participants available for analysis. At 2 h post-treatment, pain relief was achieved in 66.7% of the participants using REN versus 52.5% participants with usual care (*p* < 0.05). Pain relief at 2 h in at least one of two attacks was achieved by 84.4% of participants versus 68.9% in usual care (*p* < 0.05). REN and usual care were similarly effective for pain-free status at 2 h. The results also demonstrate the non-inferiority of REN compared with acute pharmacological treatments and its non-dependency on preventive medication use.

**Conclusion:**

REN is an effective acute treatment for migraine with non-inferior efficacy compared to current acute migraine therapies. Together with a very favorable safety profile, these findings suggest that REN may offer a promising alternative for the acute treatment of migraine and could be considered first line treatment in some patients.

**Trial registration:**

ClinicalTrials.gov NCT03361423. Registered 18 November 2017.

## Introduction

Migraine is one of the most prevalent and disabling neurologic diseases [1], characterized by recurrent, often disabling, headache attacks associated with nausea, vomiting, photophobia, and phonophobia [2]. Migraine attacks are frequently treated symptomatically with nonsteroidal anti-inflammatory drugs (NSAIDs), simple analgesics such as acetaminophen or aspirin, at times combined with caffeine, or with specific migraine treatments such as triptans and ergots [3]; however, these pharmacological treatments may be ineffective, poorly tolerated, contraindicated, and if used to excess, may lead to medication overuse headache [4, 5], migraine chronification [6] and significant medical complications. Thus, there is a significant unmet need for new, effective and well tolerated non-pharmacological acute migraine therapies.

Remote electrical neuromodulation (REN) is a novel acute migraine treatment [7], in which upper arm peripheral nerves (median and musculocutaneous) are stimulated to induce conditioned pain modulation (CPM) – a descending endogenous analgesic mechanism in which sub-threshold conditioning stimulation inhibits pain in remote body regions [8]. A recent randomized, double-blind, sham-controlled, multicenter study has demonstrated that REN provides superior, clinically meaningful relief of migraine pain and complete pain freedom at 2 h post-treatment compared to sham stimulation. Specifically, active stimulation was more effective than sham stimulation in achieving pain relief (66.7% vs. 38.8%, *p* < 0.001), pain freedom (37.4% vs. 18.4%, *p* < 0.005) and relief of most bothersome symptom (MBS) (46.3% vs. 22.2%, *p* < 0.001) at 2 h post-treatment. The pain relief and pain freedom superiorities of the active treatment were sustained for 48 h post-treatment [9]. This study also demonstrated a low incidence of device-related adverse events which was similar between treatment groups (4.8% vs. 2.4%, *p* = 0.49). All device-related adverse events, mainly reports of sensation of warmth and redness of the arm/hand, were mild, resolved within 24 h and did not require medical treatment [9].

The current report describes an exploratory, within-subject, post-hoc analysis of the aforementioned study, aiming to examine whether REN is as effective as usual care and pharmacotherapies in the acute treatment of migraine. The effect of preventative medication on REN effectiveness was also assessed.

## Methods

### Design and procedure

Exploratory within-subject post-hoc analyses were performed using data from a randomized, double-blind, sham-controlled pivotal study conducted at 12 sites (seven in the USA and five in Israel) on patients 18–75 years old who met the International Classification of Headache Disorders (ICHD 3-beta) criteria [2] for episodic migraine. The study protocol was reviewed and approved by the appropriate institutional review board for each site and all participants gave written informed consent prior to any study procedures being performed. This study was registered with ClinicalTrials.gov (NCT03361423).

Participants had 2–8 migraine headaches per month, ≤12 headache days per month, and were on either no or stable migraine preventive medications in the last 2 months prior to recruitment. The methods of the trial are fully described elsewhere [9]. Briefly, after enrollment, participants were trained to use an electronic diary, and then completed a one-month run-in phase, during which the attacks were treated according to usual care and pain scores (none, mild, moderate, or severe) were recorded at baseline and 2 h post-treatment. Eligible participants were then randomized in a 1:1 ratio to either active or sham stimulation, in a double-blind manner. Participants treated their migraine attacks with the device for 4–6 weeks (double-blind treatment phase), as soon as possible after a migraine attack began and always within 1 h of onset. Participants were instructed to avoid taking rescue medications within 2 h post-treatment. For each treated attack, participants recorded the intensity of the headache at baseline, at 2 h, and at 48 h after treatment in the app. Participants continued with their usual migraine preventive therapy and were instructed to avoid changing the dose or stopping it during the study.

### Stimulation device

The REN device (Nerivio™, Theranica Bio-Electronics Ltd., Israel) is a wireless wearable battery-operated stimulation unit controlled by a smartphone software application. The device is applied for 45 min to the lateral upper arm between the bellies of the lateral deltoid and the triceps, so that it will mainly stimulate small skin nerves. The rationale for stimulating the arm and the underlying mechanism of action are described in details elsewhere [7]. The active device produces a proprietary electrical signal comprising a modulated symmetrical biphasic square pulse with a modulated frequency of 100–120-Hz, pulse width of 400 μs, and up to 40 mA output current (adjusted by the participant). Although the pulse stimulates C and Aδ noxious sensory fibers above their depolarization thresholds, the stimulation energy is low enough to maintain the overall sensory experience below perceptual pain threshold.

### Participants (subpopulation for post-hoc analysis)

Within-subject comparisons between REN and usual care or pharmacological treatments were performed on a subgroup of participants who treated at least one attack with the active REN device and reported pain intensity at 2 h post-treatment. The effect of preventive medications on the effectiveness of REN was also investigated in this population. Additional analyses were performed on participants who had treated at least two attacks (and reported pain intensity at 2 h post-treatment) in the run-in phase and treated at least two attacks (and reported pain intensity at 2 h post-treatment) with the active REN device in the double-blind treatment phase.

### Data analysis

REN pain relief and pain-free responses at 2 h post-treatment were compared to the response of individual usual care which included migraine specific and non-specific pharmacological treatments and no pharmacological treatment) and to the responses of pharmacological treatments (specific and non-specific treatments). The evaluation included: (1) a comparison between response rates of REN and usual care or pharmacological treatments in a single treatment (comparing the first attack in the run-in phase and the first attack in the double-blind treatment phase following a training treatment [i.e., test treatment]) [9]; and (2) a comparison between response rates of REN and usual care or pharmacological treatments in at least 1 of 2 attacks (comparing the response rate in the first two attacks in the run-in phase with the response rate of the training and test treatment in the double-blind treatment phase). All analyses were performed on fully treated (30–45 min) attacks preceded by at least 48 headache-free hours. Efficacy data were compared using the McNemar’s test, McNemar-Bowker test of symmetry or chi-squared test, as appropriate, with SPSS Statistics v20.0 (IBM corporation). All statistical tests were 2-tailed, with statistical significance set at *p* < 0.05. No adjustments were made for multiple comparisons. All authors had full access to all study data.

## Results

### Participants

The first patient was enrolled in December 2017, and the last patient completed the double-blind phase of the study in October 2018. Of the 296 participants enrolled to the study, 252 were randomized to active and sham groups. Ninety-nine participants completed a test treatment with the active REN device within 1 h of headache onset and reported pain level at 2 h. Of the 99 participants, 34 indicated they use preventive medications. 90 of the 99 participants had treated at least two attacks with pain intensity reported at 2 h post-treatment in the run-in phase and at least two attacks with pain intensity reported at 2 h post-treatment with the active REN device. The demographic and clinical characteristics of the 99 participants who treated at least one attack with REN were similar to those of the entire study population [9]; 80.6% were women with a mean age of 43.8 ± 12.25 years.

### Baseline characteristics of migraine attacks

The baseline characteristics of migraine attacks treated with usual care in the run-in phase and the attacks treated with REN are depicted in Table 1. The characteristics of the first treated attack in the run-in phase (i.e., attacks treated with usual care) were similar to those of the test treatment with active REN (pain severity: *p* = 0.444; photophobia: *p* = 1.00; phonophobia: *p* = 0.286), except for nausea which was more frequently reported in the test treatment compared to the first attack treated with usual care (*p* = 0.035). Generally, the characteristics of treated migraine headaches were comparable to those reported in previous migraine studies [10–12] and are consistent with the pain intensity characterization of the target population of the device [13].

**Table 1:** Baseline characteristics of the treated attacks

Characteristic	1st attack in the run-in phase	2nd attack in the run-in phase^a^	Training treatment	Test treatment
Baseline pain severity, % (n/N)				
Mild	46.5% (46/99)	33.3% (33/98)	54.5% (54/99)	35.4% (35/99)
Moderate	49.5% (49/99)	49.5% (49/98)	40.4% (40/99)	57.6% (57/99)
Severe	4.0% (4/99)	16.2% (16/98)	5.1% (5/99)	7.1% (7/99)
Presence of baseline associated symptoms, % (n/N)				
Nausea	14.1% (14/99)	29.3% (29/98)	24.2% (24/99)	25.3% (25/99)
Photophobia	64.6% (64/99)	72.7% (72/98)	64.6% (64/99)	63.6% (63/99)
Phonophobia	61.6% (61/99)	58.6% (58/98)	50.5% (50/99)	55.6% (55/99)

^a^One participant reported only one attack in the run-in phase

### REN versus usual care

**Fig. 1:**
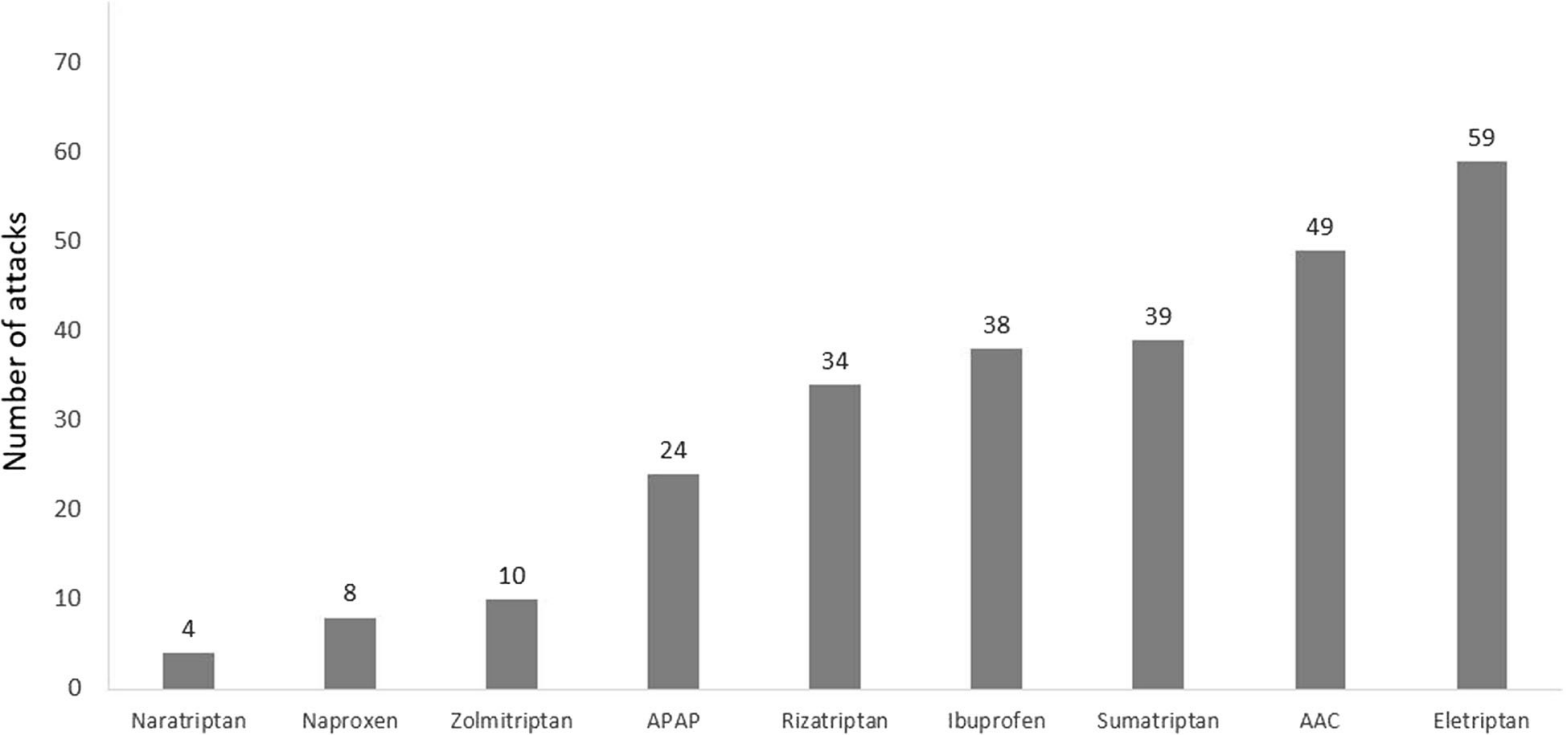
Number of participants using different types of acute pharmacological treatments in their first reported attack in the run-in phase. AAC, aspirin, acetaminophen and caffeine; APAP, acetaminophen

Usual care included specific acute migraine medications (e.g. triptans), non-specific acute medications (e.g. NSAIDS and acetaminophen) and no pharmacological treatments (data on non-pharmacological treatments that could have been used, such as biofeedback, was not collected). Sixty-nine participants used acute medications in their first reported attack in the run-in phase, of which 31 participants used specific acute migraine medications and 38 participants used non-specific acute medications. Figure 1 presents the number of patients taking each type of pharmacological treatment.

**Fig. 2:**
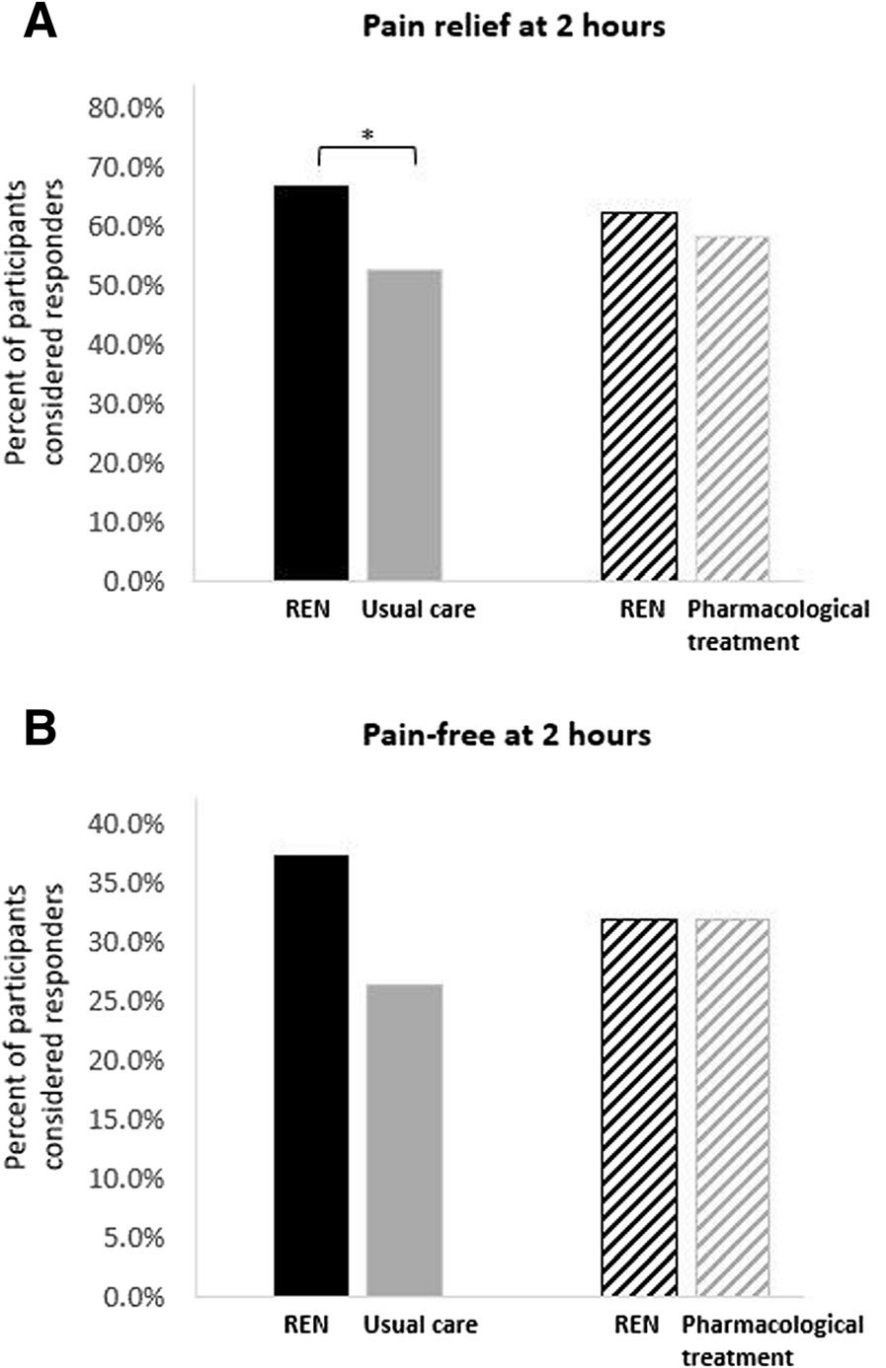
Efficacy comparison of pain responses in a single attack. **a** Pain relief at 2 h post-treatment of REN (solid black and diagonal black) compared with usual care (solid gray) and pharmacological treatment (diagonal gray). **b** Pain-free at 2 h post-treatment of REN (solid black and diagonal black) compared with usual care (solid gray) and pharmacological treatment (diagonal gray). *p < 0.05

Pain relief and pain-free responses at 2 h post-treatment of the first attack reported in the run-in phase were compared with REN responses in the test treatment in the double-blind treatment phase. The percentages of participants achieving pain relief at 2 h were 66.7% (66/99) for REN treatment and 52.5% (52/99) for usual care (*p* = 0.034; Fig. 2a). The percentages of participants achieving pain freedom at 2 h were 37.4% (37/99) for REN treatment and 26.3% (26/99) for usual care (*p* = 0.099; Fig. 2b).

**Fig. 3:**
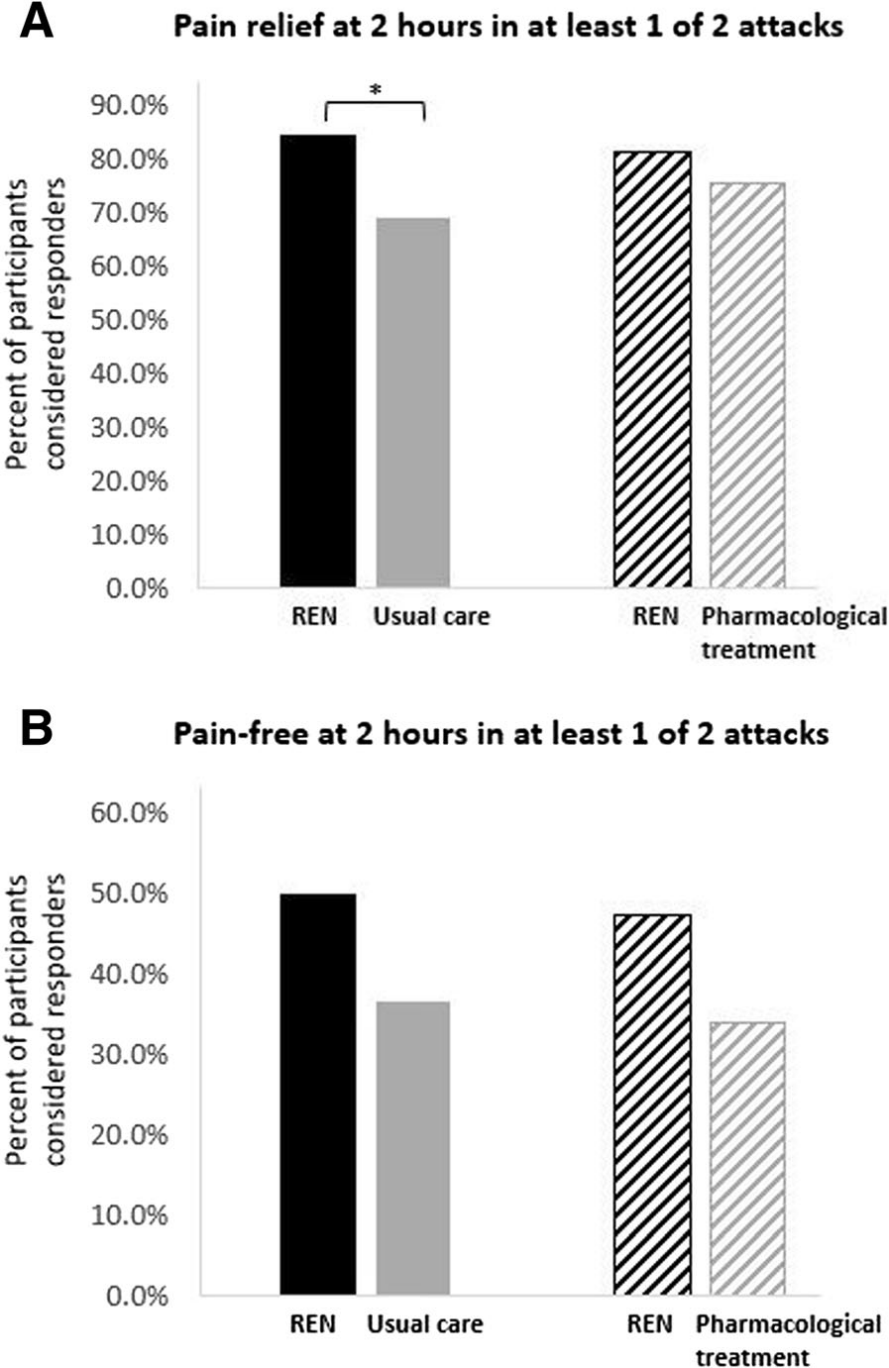
Efficacy comparison of pain responses in at least 1 of 2 attacks. **a** Pain relief at 2 h post-treatment in at least 1 of 2 attacks following REN treatment (solid black and diagonal black) compared with responses usual care (solid gray) and pharmacological treatment (diagonal gray). **b** Pain-free at 2 h post-treatment in at least 1 of 2 attacks following REN treatment (solid black and diagonal black) compared with usual care (solid gray) and pharmacological treatment (diagonal gray). *p < 0.05

For the 90 participants who treated at least two attacks in both the run-in and the double-blind treatment phases, pain relief and pain-free responses at 2 h post-treatment in at least one of two attacks in the run-in phase were compared with REN responses at 2 h in at least one of two attacks. The percentages of participants achieving pain relief at 2 h in at least one of two attacks were 84.4% (76/90) for REN treatment and 68.9% (62/90) for usual care (*p* = 0.02; Fig. 3a). Pain freedom at 2 h in at least 1 of 2 attacks was achieved by 50.0% (45/90) of the participants with REN treatment versus 36.7% (33/90) with usual care (*p* = 0.050; Fig. 3b).

### REN versus acute migraine pharmacological treatments

Similar comparative analyses of a single attack and response in one of two attacks were performed on subgroups of 69 participants who used acute medications to treat the first attack reported during the run-in phase and 53 participants who treated both attacks in the run-in phase with acute medication, respectively. Of the 69 participants, 62.3% (43/69) achieved pain relief at 2 h following REN treatment compared with 58.0% (40/69) for pharmacological treatments (*p* = 0.690; Fig. 2a). The percentages of participants achieving pain freedom at 2 h were 31.9% (22/69) for both REN treatment and pharmacological treatments (*p* = 1.00; Fig. 2b).

In people who treated both attacks in the run-in phase with acute medication (*N* = 53), the percentages of participants achieving pain relief at 2 h in at least one of two attacks were 81.1% (43/53) for REN treatment and 75.5% (40/53) for pharmacological treatments (*p* = 0.607; Fig. 3a) and the percentages of participants achieving pain freedom at 2 h in at least one of two attacks were 47.2% (25/53) for REN treatment and 34.0% (18/53) for pharmacological treatments (*p* = 0.143; Fig. 3b).

### Non-dependency of REN effectiveness on use of preventive treatment

An additional aim of these post-hoc analyses was to evaluate the efficacy of REN as function of preventive medication use. The subgroup of 99 participants who completed a test treatment with the active REN device within 1 h from symptom onset and reported pain level at 2 h was retrospectively classified to preventive medication users (*N* = 34) and non-users (*N* = 65) based on their migraine history assessment. Pain relief rates of REN treatment were similar in people who use preventive migraine treatments (67.6% [23/34]) compared with people who do not use preventives (66.2% [43/65]; *p* = 0.881). Similarly, pain-free rates were similar in people who use preventive migraine treatments (41.2% [14/34]) compared with people who do not use preventives (35.4% [23/65]; *p* = 0.572).

## Discussion

The overall results demonstrate that REN is an effective acute treatment for migraine with non-inferior efficacy to usual care in general and to acute migraine pharmacological treatments specifically. REN, when compared to usual care or pharmacological treatments, proved to be as effective for pain relief and pain-free at 2 h post-treatment both in a single attack and across two attacks. Importantly, the efficacy of REN treatment did not depend on preventive medications use.

The significant need for new effective and safe acute treatments of migraine has led to the development of REN [7]. A recent study has demonstrated favorable efficacy and safety outcomes of REN [9], with a superior efficacy compared to other neuromodulation devices for acute migraine treatment [10, 14]. This study has also shown that REN has a favorable tolerability profile compared with triptans [15] and with new pharmacological agents, such as centrally acting serotonin (5-HT_1F_) agonists that lack vasoconstrictor activity [16]. The within-subject pos-hoc analysis presented in this paper extends these findings by providing direct evidence that the efficacy of REN is non-inferior to individual usual care and to current acute migraine pharmacological treatments. It appears that, at least in some patients, REN treatment may be even more efficacious than usual treatment in terms of pain relief at 2 h post-treatment. Further investigation is necessary to better characterize this population.

The current analysis also suggests that the efficacy of REN does not depend on whether or not patients are taking preventive medications. Therefore, REN may be effective for a wide range of people with migraine, as opposed to the single-pulse transcranial magnetic acute migraine neuromodulation device in which use of concomitant preventive drugs could affect its effectiveness [14].

In the context of this study, usual care (which included either no pharmacological treatment or migraine specific and non-specific pharmacological treatments) and pharmacological treatment (which included any type of acute medication) provided a window into real-life management and experiences without producing expectations about the efficacy of a particular treatment approach. Although direct comparisons between this exploratory post-hoc analysis and randomized clinical trials must be made cautiously, pain relief and pain-free response rates across pharmacological treatments in the current study are in the same range as those reported in previous studies [17, 18]. This suggests that our data reflects the clinical efficacy of pharmacological treatments observed in other randomized trials and that the results of the current study could be generalizable. A prospective head to head trial is required to further investigate this.

The current study has several limitations. First, this paper presents a post-hoc analysis and not a prospective head to head trial. Second, the small sample size precludes the comparison between different classes of pharmacological treatments (e.g. NSAIDS, acetaminophen, triptans) and thus all drugs were classified to one group of pharmacological treatments. This approach is supported by a review of comparative clinical trials of acute migraine treatments, indicating that in a clinical trial setting, the efficacy of migraine specific and non-specific acute mediations is comparable [17]. Finally, our dataset of multiple attacks included different pharmacological treatments (or no treatment) for a single person, which decreases its scientific purity; however, this intra-individual variability encompasses real-life migraine management, varying across attacks within the same patient, thus empowering our findings. Notably, while the post-hoc analysis presented in the current paper focused on a comparison between current pharmacological treatments versus REN, in real life, it is highly likely that a combined pharmacological and REN therapy will have an added benefit since the two treatment modalities are independent and should be compatible.

## Conclusions

REN is an effective acute treatment of migraine attacks, with non-inferior efficacy compared with usual care and various pharmacological treatments in this analysis. Along with its favorable safety and tolerability profiles and its non-pharmacological nature that alleviates the adverse events and medical risks associated with some current migraine pharmacological treatments, our findings suggest that REN may be useful as an alternative or adjunctive acute migraine treatment.

## Data Availability

The data may be available upon reasonable request.

## Abbreviations

CPM: Conditioned pain modulation
NSAIDs: Nonsteroidal anti-inflammatory drugs
REN: Remote electrical neuromodulation
